# Perspective: Personalized Management of Oxidative and Nitrosative Stress in Post-Exercise Recovery with a Particular Emphasis on the Potential of Micro-Immunotherapy

**DOI:** 10.3390/sports14060239

**Published:** 2026-06-09

**Authors:** Camille Jacques, Ilaria Floris

**Affiliations:** Preclinical Research Department, Labo’life France, Pescalis-Les Magnys, 79320 Moncoutant-sur-Sevre, France; ilaria.floris@labolife.com

**Keywords:** eustress, distress, micro-immunotherapy, cytokines, mTOR, redox status, post-effort recovery, sport, mitochondria health, immunomodulation

## Abstract

The understanding of oxidative stress is being refined leading to the use of the terms “oxidative distress” and “eustress”. This reflects the dual role of reactive oxygen species (ROS) and reactive nitrogen species (RNS) in both pathology and physiology, emphasizing the complexity of the mechanisms influencing the redox status. This review discusses how these redox mechanisms interact with key signaling pathways, specifically the mammalian/mechanistic target of rapamycin (mTOR) and peroxisome proliferator-activated receptor-gamma coactivator (PGC-1α), which are crucial for mitochondrial health and muscle recovery. During exercise, the contraction of skeletal muscles increases ROS production which, through redox signaling, triggers mitochondrial biogenesis, enhances the antioxidant defenses and stimulates glucose metabolism, contributing to cardiovascular function and health. There is a large consensus about the importance of physical exercise in maintaining the redox homeostasis. However, the redox status could be disturbed after an intense and/or long physical effort, and signs such as markers of oxidative distress were identified. In that context, antioxidant strategies are warranted to prevent oxidative damage and help recovery. Given the many factors influencing the redox status of the body, including the training status, the duration and type of exercises and effort, diet, lifestyle, genetic polymorphisms, and circulating cytokines, a personalized approach is necessary. Targeted therapeutic interventions become important for preventing oxidative damage and helping recovery. In this review, we discuss the potential benefits of micro-immunotherapy (MI), as a multi-target approach utilizing signaling molecules, including cytokines at low doses (LD, typically 3–5 centesimal Hahnemannian CH dilutions) and ultra-low doses (ULD, from 6 CH upwards). We focused specifically on the investigational MI medicine 2LMIREG, and propose its application in preventing oxidative distress and restoring redox balance. Additionally, this review explores how the redox status interplays with the immune system, presenting preclinical data on 2LMIREG as a proof-of-concept for a tailored immunoregulatory strategy to enhance both immune and oxidative adaptations.

## 1. Introduction

It is now well established that engaging in regular moderate exercise has many health benefits, as it can lower the risk of disease associated with disturbed metabolism, certain cancers and bone fractures. It is advised that all adults aged 18 and over engage in at least 150 min of moderate-intensity aerobic physical activity weekly, which should result in a moderate increase in heart rate, or alternatively, at least 75 min of vigorous-intensity activity each week, which involves a significant increase in pulse rate [1]. While these moderate exercise guidelines provide a foundation for maintaining health, it is equally important to consider how varying intensities of exercise can lead to different physiological impacts. Indeed, on the other hand, enduring intense exercise sessions can lead to heightened systemic inflammation, temporarily weakening certain components of the immune system, such as neutrophil activity and lymphocyte function, with these effects generally lasting between 3 and 24 h after exercising, depending on the duration and the intensity [2]. This transient immunosuppression is often exacerbated by the excessive production of reactive oxygen species (ROS), leading to oxidative distress which further impairs immune cell function. Indeed, intense physical activity is associated with high increases in ROS production, muscle damage [3], inflammation, as well as increased lactate buildup [4]. When the generation of free radicals, including ROS and reactive nitrogen species (RNS), surpasses the cells’ ability to eliminate them through their antioxidant defense mechanisms, numerous harmful effects on cellular metabolism occur.

This review focuses on understanding the role of ROS-related pathways, and, to a lesser extent, of RNS-related pathways in post-exercise recovery, with the aim of developing optimized, personalized strategies, including immunomodulatory interventions, for preventing oxidative damage. In particular, the main cellular antioxidant mechanisms and the potential interactions between cytokines and these antioxidant systems will be investigated. Moreover, crucial pathways such as mammalian/mechanistic target of rapamycin (mTOR) and peroxisome proliferator-activated receptor-gamma coactivator (PGC-1α) will be investigated to elucidate their interactions with ROS and their implications for muscle recovery. By integrating non-exhaustive data from controlled laboratory studies and “real-world” data from trained persons, this review aims to uncover the complex biological processes influenced by ROS/RNS. Moreover, the role played by cytokines on these pathways and the antioxidant system will be investigated, with the aim of assessing the potential of cytokine-based immunomodulatory strategies to develop optimized recovery strategies. These strategies must account for the multifactorial mechanisms (including the training status, the duration and type of exercises and effort, diet, lifestyle, genetic polymorphisms, and circulating cytokines) driving optimal adaptations, and recovery. Thus, personalized approaches and targeted therapeutic interventions should be tailored to help the body in restoring the redox status. In this context, immunomodulatory strategies such as micro-immunotherapy medicines (MIMs), by employing signaling molecules such as cytokines and growth factors at low doses (LD) and ultra-low doses (ULD), hold potential. For the sake of complete transparency, the authors disclose their employment by Labo’life France, a company service provider specialized in preclinical research and regulatory affairs of the group Labo’life, which manufactures and commercializes the MIM discussed herein. Thus, this review aims to explore the concept and potential application of such MI medications, and particularly 2LMIREG, as a novel approach for immunomodulation in the context of post-exercise recovery. The primary objective is to examine how MI could mitigate exercise-induced oxidative and nitrosative stress while modulating immune cells, offering a promising alternative to current antioxidant-based supplementation strategies for recovery. While clinical studies are not yet available, this review seeks to evaluate MI’s potential based on existing in vitro data, providing a proof-of-concept for its practical use in enhancing recovery outcomes in the future. By addressing these aspects, the review aims to fill existing knowledge gaps regarding the interplay between oxidative stress, immune function, and recovery, guiding researchers and practitioners to consider MI’s potential benefits. In particular, the preclinical data published so far on 2LMIREG are presented in the last sections of the review and the perspectives of this medicine are discussed in the framework of post-effort recovery in sports.

The structure of the review is as follows: Section 2 defines the dual role of ROS and the concept of oxidative distress and oxidative eustress; Section 3, Section 4 and Section 5 investigate the redox system during physical exercise, and the crucial roles of the mammalian/mechanistic target of rapamycin (mTOR) and peroxisome proliferator-activated receptor-gamma coactivator (PGC-1α) pathways in muscle recovery and discuss how excessive ROS production reduces performance and delays recovery. Section 6 and Section 7 explore the diverse factors modulating redox homeostasis and how cytokines could be used as an antioxidant strategy, especially when used at ULD in MIM. Finally, Section 8 and Section 9 focus on the potential of MI, and especially 2LMIREG, in the framework of post-effort recovery in sports.

## 2. The Dual Role of Reactive Oxygen Species and the Concept of Oxidative Distress and Oxidative Eustress

### 2.1. From Biochemical Background…

- Reactive oxygen species (ROS) regulate many physiological processes such as host defense, hormone production or muscle adaptation to effort [5], for instance. This first section will serve as an introduction on ROS and their biogenesis. As a first definition, an atom or molecule with one or more unpaired electrons that can exist independently is commonly known as a “free radical” [6]. Thus, ROS are chemically reactive molecules containing oxygen, which play a dual role in physiological processes [7]. These molecules can arise either intrinsically, from the oxidative phosphorylation (OXPHOS) process or externally, in response to xenobiotics and pollution. When generated through biological systems, they are typical byproducts of mitochondrial metabolism and encompass: (1) the short-lived oxygen free radicals like the superoxide anion radical (O_2_·−), the hydroxyl radical (·OH), or nitric oxide (NO), among others, or (2) non-radical compounds, such as hydrogen peroxide (H_2_O_2_), which displays a comparatively extended biological lifespan (cellular half-life of approximately 1 ms) [8]. Hydroxyl radicals, ·OH, because of their high reactivity, cannot permeate membranes, and are thus considered to be one of the most damaging ROS amongst the biological systems [9]. Furthermore, nitrosative damage is primarily caused by RNS such as nitric oxide (NO) and peroxynitrite (ONOO^−^), which, when overproduced, can lead to protein modification (e.g., nitration of tyrosine residues), DNA damage, and disruption of cellular signaling, which are implicated in various pathological conditions, including inflammatory diseases and degenerative diseases [10]. Both types of damage can disrupt cellular homeostasis and are involved in the pathophysiology of several diseases. However, they differ primarily in the types of reactive species involved and their specific biochemical interactions. From a molecular standpoint, and as nicely explained in the review from Mittal et al., O_2_·− generated by mitochondria are dismutated by manganese superoxide dismutase (MnSOD) in the mitochondrial matrix to produce H_2_O_2_. Indeed, as MnSOD catalyzes the conversion of two superoxide anions into oxygen and H_2_O_2_, it plays a critical role in mitigating oxidative stress within the mitochondria [11]. This H_2_O_2_ can then traverse the mitochondrial outer membrane to affect targets in the cytosol. As a result, this process can lead to various functional outcomes, including the activation of redox-sensitive transcription factors like hypoxia inducible factor (HIF)-1α and nuclear factor kappa-B (NF-κB), the induction of pro-inflammatory cytokines, and the stimulation of inflammasomes. Interestingly, the endoplasmic reticulum has also been shown to be a high H_2_O_2_-producing organelle, in response to its anabolic activity of oxidative protein folding [8]. Finally, it is also worth mentioning that the byproducts of nitrosative distress can have a repercussion on key enzymes of energy metabolism, leading to their inactivation [12].

### 2.2. … To Biological Paradox

Reactive oxygen species are also involved in the initiation and progression of pathological processes, such as chronic diseases [13,14]. Regarding the duality of the effects of ROS and their “biological paradox”, as both toxic components and signaling molecules, a controlled regulation of their production is vital for maintaining the integrity of living organisms. The comprehension of the pathways responsible for regulating ROS homeostasis is essential, not only for reducing ROS toxicity, but also, to offer compelling evidence for the specificity of their signaling. For these reasons, the concept of oxidative stress is being recently refined, and the terms oxidative “distress” and “eustress” are employed to differentiate whether we are talking about a physiological adaptation aiming at a “physiological” control of the redox status (oxidative eustress), or a truly disrupted redox signaling (oxidative distress), associated with cell damage and diseases [15,16].

Oxidative eustress represents the transient increase in ROS and RNS levels, which in the context of exercise, act as a signaling trigger promoting beneficial effects such as mitochondrial biogenesis, the enhancement of antioxidant defenses, and the improvement of muscle functions. Conversely, oxidative distress results from a chronic, excessive, or overwhelming production of ROS, such as during short recovery periods, that surpasses the cell’s antioxidant capacity, leading to damage to lipids, proteins, and nucleic acids, ultimately hindering muscle performance and recovery. To clinically distinguish these two states, specific biomarkers associated with cellular damage and lipid peroxidation, such as plasma protein carbonyl levels, and other parameters discussed in the following sections, could be monitored. This review highlights the importance of differentiating between the two states and of intervening with a personalized approach during post-exercise recovery, and a particular emphasis has been placed on the potential benefits of MI.

## 3. The Redox System During Physical Exercises

### 3.1. The Redox System Is Influenced by Aerobic Training and Resistance Training Modalities in Healthy People and in People with Metabolic Disorders

This section explores how aerobic and resistance training affect the redox system, and the need to explore these effects through appropriate experimental models and clinical trials. It then examines training adaptations for long-term redox balance improvement, and introduces the concept of hormesis. Lastly, it highlights differences between healthy individuals and those with metabolic disorders, offering insights into their distinct physiological responses.

(a) Acute exercise effects and long-term adaptations: a need for clinical trials and experimental models

During exercise, the production of ROS is intensified due to several factors including increased mitochondrial respiration and activation of various enzymatic sources such as NADPH oxidase. While acute increases in ROS production act as signaling triggers to promote adaptive response and improve muscle function [17], chronic excessive ROS production post-exercise can lead to oxidative stress, potentially resulting in cellular damage and impaired recovery, as has been shown in over-trained athletes [18].

The balance between ROS production and the body’s antioxidant defense systems is crucial in mitigating oxidative damage and enhancing the positive outcomes of exercise training. Understanding the mechanisms regulating ROS production and the subsequent effects on muscle adaptation and performance is essential for optimizing training protocols and recovery strategies. The exploration of ROS in the context of post-exercise recovery is a dynamic field that can be investigated through clinical trials and experimental models, including in vitro and in vivo studies. In clinical settings, monitoring ROS levels and antioxidant capabilities, especially in athletes, is crucial. This assessment, alongside controlled exercise regimens, provides insights into how these factors can relate to performance and recovery.

(b) Training adaptations: the hormesis concept

Thus, the concept of exercise-induced ROS playing a crucial role in the body’s adaptation to oxidative stress through the “hormesis” phenomenon has emerged, highlighting the notion that antioxidant capacity and mitochondrial biogenesis could be enhanced without necessarily increasing oxidative stress markers [19]. Such plasticity of the oxidant systems has been highlighted, for instance, by Zabet et al., who reported that resistance training with moderate and progressive intensity could lead to improvements in oxidative stress adaptability, as indicated by changes in biomarkers such as serum 8-isoprostane and reduced glutathione levels [20]. Even if the significance of the results was only demonstrated for the serum 8-isoprostane, the study concluded that periodic increases in resistance training load correlated with beneficial enhancements of oxidative stress markers, suggesting a protective adaptation process. Regarding these results, it could be hypothesized that there is a possible more pronounced sensitivity of lipid peroxidation markers to resistance training-induced oxidative changes. This could indicate that the body tends to prioritize the modulation of oxidative damage at the lipid level, possibly due to the immediacy of lipid peroxidation in cellular membranes, while maintaining stable glutathione levels to ensure a consistent and robust baseline antioxidant defense. Such a response exemplifies the body’s strategic adaptation, emphasizing repair and signaling over clear enhancement of antioxidant reserves. Again, these possibilities emerge from one study and more research, assessing multiple markers, is still needed. Similarly, Vincent et al. demonstrated that, in overweight and obese older adults, resistance training reduced exercise-induced oxidative stress along with lowering homocysteine levels, highlighting its potential to mitigate oxidative stress-related risks in this population [21].

Interesting observations have emerged regarding the intensity and duration of the training. While moderate training shows a beneficial effect on oxidative stress, the intensity of the exercise can lead to varying outcomes [22]. For instance, a study conducted in rats noted that increased exercise intensity elevated plasma protein carbonyl levels, a marker of oxidative stress, indicating a level of oxidative damage while simultaneously enhancing total plasma antioxidant capacity [23]. On the other hand, incremental exhaustive exercise did not change oxidative stress markers when directly assessed in the muscles. This illustrates a nuanced relationship where low to moderate resistance training can produce adaptive signals favorable for reducing oxidative stress, whereas excessively high intensities can lead to oxidative damage. In addition, the tissue-specific nature of oxidative stress responses to exercise, as observed in this rat study, merits further discussion and may suggest a nuanced balance between systemic and local adaptations. The discrepancy between plasma and muscle markers may reflect the body’s prioritization of systemic oxidative stress management over localized muscle adaptations. For instance, plasma markers could indicate a broader, circulating oxidative environment, potentially influenced by systemic factors such as cortisol or systemic immune activation in response to exercise. Conversely, muscle tissue may exhibit resilience or adaptive capacity owing to regular exposure to oxidative stress and the presence of localized antioxidant mechanisms. This differential response could imply that systemic oxidative stress might benefit from strategies focused on systemic recovery and modulation, such as nutritional antioxidants or anti-inflammatory interventions, which may imply the use of immunotherapeutic modulators, whereas local muscle stress may rely more on intrinsic recovery processes, enhanced by rest and gradual training adaptations tailored to reinforce muscle-specific antioxidant systems. Understanding these dynamics may allow for more targeted recovery strategies that optimize both systemic and muscle-specific oxidative balance and recovery.

Moreover, it is essential to differentiate the effects of resistance training from those of aerobic training regarding oxidative stress levels. While cardiovascular exercises generally tend to induce higher oxidative stress due to prolonged increases in oxygen consumption rates, resistance training appears to induce a more balanced oxidative profile that encourages adaptation without overwhelming cellular defenses [24,25]. This distinction emphasizes the importance of integrating diverse exercise modalities when considering interventions aimed at managing oxidative stress.

(c) Population differences: healthy vs. metabolic disorders

Resistance training’s positive impact on oxidative stress is particularly relevant in populations with metabolic disorders, including type 2 diabetes. El-Kader et al.’s work illustrated that structured resistance exercise programs significantly improved oxidative stress among obese individuals with type 2 diabetes, demonstrating ameliorations regarding insulin sensitivity and metabolic health [26]. Additionally, Vinetti et al. provided evidence that supervised exercise training, incorporating both resistance and aerobic components notably reduced oxidative stress while improving cardiometabolic risk factors [27]. Of note, while the endurance training protocols of this study involved cycling on mechanically braked cycle ergometers, the resistance training part consisted of different exercises involving the major muscle groups (upper limb, lower limb, chest, back and core), worked both in calisthenics and in repetitions with ankle weights, dumbbells and elastic bands (in three sets of eight repetitions, progressively improved to three sets of 12–15 repetitions). This relationship underscores the therapeutic potential of incorporating resistance training in strategies aimed at managing oxidative stress-driven conditions, such as metabolic disorders.

Resistance training is a potent form of exercise that holds considerable promise in managing oxidative stress. By fostering adaptive changes associated with increased antioxidant capacity and balanced ROS levels, resistance training can serve as a protective intervention in promoting long-term health and reducing oxidative stress-related risks. Future recommendations may include tailored resistance training regimens that balance intensity and duration to maximize the protective benefits, while minimizing potential oxidative damage.

### 3.2. Resistance Training Is Beneficial for Mitochondria Health

When assessed by transmission electron microscopy, 75–85% of the skeletal muscle cells’ volume is occupied by myofibrils, the contractile material responsible for their main cellular functions. However, assessment of mitochondrial volume density in muscle fibers determined that untrained skeletal muscle displayed a mitochondrial density of 2–6%, and that this density was rising to 11% in trained athletes [28], highlighting the plasticity of these cells, and the importance of these organelles for performance. In addition, from a molecular standpoint, as signaling pathways such as mTOR and PGC-1α are involved in muscle function and converge on the mitochondria, a link can definitely be made between resistance training and mitochondria health, within the muscles. As mentioned above, resistance training has been shown to improve mitochondrial adaptations, directly within skeletal muscle [29]. For instance, a study assessed skeletal muscle mitochondrial respiration in a group of resistance-trained athletes compared to untrained controls [30]. The resistance-trained athletes exhibited higher coupled mitochondrial respiration, which is adenosine triphosphate (ATP)-producing, per milligram of muscle. Additionally, the respiratory control ratio for adenosine diphosphate (ADP) was also greater in these athletes, suggesting that the capacity of ATP production within the muscles is more important with resistance-training. Other studies reported the effects of resistance training on mitochondrial parameters, and, among these, Porter et al. assessed the qualitative and quantitative changes in skeletal muscle mitochondrial respiration after 12 weeks of resistance training in eleven healthy men, by high-resolution respirometry [31]. Maximal coupled OXPHOS with electron input from both complex I and II of the electron transport chain increased by 1.4 times following resistance exercise training. In this study, no impact was found on skeletal muscle citrate synthase activity, nor on the abundance of complex II, III and IV of the electron transport chain, or on complex V. However, the protein expression of complex I was found to be increased by 11%. In the same way, when assessed in the context of disease, Gorgey et al. showed that skeletal muscle hypertrophy could potentially improve mitochondrial bioenergetics following spinal cord injury (SCI). Indeed, they found that neuromuscular electrical stimulation-resistance training was likely to boost the activity of complex III in vastus lateralis biopsies from sedentary individuals with an SCI [32]. Interestingly, and to better illustrate the intricate connection between mitochondrial health and ROS production, the study from Hashimoto et al. provided evidence on how training increases the capacity for lactate clearance via oxidation [33]. Indeed, their work allowed for a better understanding of how, during physical activity, temporary increases in lactate concentration in tissues and oxygen consumption in mitochondria lead to ROS generation. This, in turn, triggered a transcriptional network that signaled adaptive cellular responses, which included enhancements in the lactate oxidation complex, the creation of new mitochondria, and the upregulation of antioxidant enzymes and components involved in calcium signaling.

Here it can be concluded that the observed mitochondrial adaptations, such as increased OXPHOS capacity and enhanced protein expression of key complexes within the electron transport chain, highlight the profound influence of resistance training on cellular energy systems. These adaptations are closely intertwined with the activation of critical signaling pathways, notably mTOR and PGC-1α, which orchestrate a broad range of cellular processes including energy metabolism, muscle growth, and antioxidant responses. The enhancement of mitochondrial function through training is not solely a matter of isolated biochemical changes; it reflects an integrated and dynamic response facilitated by these pathways. mTOR, as a master regulator coordinating anabolic and catabolic balance in muscle cells, and PGC-1α, as a pivotal coactivator in mitochondrial biogenesis, collectively drive the cellular restructuring that underpins improved bioenergetics and resilience. Together, they illustrate how exercise-induced stress and physiological demands trigger a sophisticated network of signaling pathways, promoting optimal mitochondrial health and systemic adaptation.

## 4. Mitochondria, mTOR and PGC-1α Pathways

Resistance training not only plays a crucial role in enhancing muscle hypertrophy but also significantly influences mitochondrial health through the activation of key molecular pathways, primarily the mTOR and PGC-1α pathways. Research has shown that resistance exercise activates mTOR signaling, which is critical for protein synthesis and muscle growth, and additionally regulates mitochondrial biogenesis and function [30,34].

### 4.1. Mammalian/Mechanistic Target of Rapamycin (mTOR) Pathway: Muscle Growth, Mitochondria Health and Immunity

The mammalian/mechanistic target of rapamycin (mTOR) is a 289 kDa serine/threonine kinase conserved across evolution, responsible for regulating various cellular functions such as cell division and protein synthesis, and whose activity is influenced by nutrient availability and growth factors [35,36]. In the context of this review, it is crucial to pinpoint that, as mTOR finely tunes the anabolic/catabolic processes within the muscle cells, it acts as a master regulator of muscle growth/maintenance [37]. It is now understood that the mTOR pathway is the primary nutrient-sensitive regulator of growth in animals [38], and plays a pivotal role in physiology, metabolism, aging, and prevalent diseases. The mTOR protein engages with various other proteins to create two separate multiprotein complexes: mTOR complex 1 (mTORC1) and mTOR complex 2 (mTORC2), each of which has differing sensitivity to rapamycin [39].

Alterations in mitochondrial respiration rates are linked to modifications in mitochondrial mass, fission and fusion rates, mitochondrial biogenesis and mitophagy, in addition to changes in mitochondrial DNA (mtDNA) copy number, transcription, and translation. Recently, a growing body of evidence has highlighted the role of mTORC1 as a key regulatory hub in these processes, managing energy usage by the translation apparatus and ATP production within the mitochondria [40]. mTORC1 plays a massive role in metabolic flexibility as its activation facilitates the translation of metabolic enzymes essential for proliferation [41]. It also promotes the production of nuclear-encoded mitochondrial proteins, components of mitochondrial complexes I and V, and the synthesis of transcription factors like myelocytoma (Myc) and HIF-1α, which are crucial for metabolic reprogramming.

Finally, to put in perspective the fact that the mTOR pathway is an important node in the cellular processes leading to post-effort recovery, it is worth highlighting the relationship between ROS and the mTOR signaling. The impact of ROS on the mTOR pathway is dependent on the dose and duration of exposure. Specifically, low doses of ROS exposure activate mTORC1, promoting adaptive responses. Conversely, high concentrations or prolonged ROS treatment result in decreased mTORC1 activity. Indeed, it is important to keep in mind that ROS can either inhibit or activate the mTOR pathway through various mechanisms, while mTOR signaling can increase oxidative stress by downregulating the expression of antioxidant enzyme genes [42]. In addition, it has been demonstrated that H_2_O_2_-mediated oxidative stress impairs mTOR-mediated phosphorylation of ribosomal p70 S6 kinase (S6K1) and eukaryotic initiation factor 4E (eIF4E) binding protein 1 (4E-BP1) [43]. Li et al. also documented that low doses of ROS exposure activate mTORC1, whereas high concentrations or prolonged ROS treatment result in decreased mTORC1 activity both in vivo and across various cell lines [44]. In line with these results about H_2_O_2_-mediated oxidative stress, it is also interesting to mention that the accumulation of ROS/RNS was also reported to inhibit mTORC1 activity [45], another potential mechanism though which nitrosative stress could limit protein synthesis after strenuous exercise.

Interestingly, the regulation of metabolism through mTORC2 plays a role in the polarization of F-actin at the leading edge of the cells [46], and was directly associated with the mitochondrial-associated membrane (MAM), and was crucial for maintaining MAM integrity and function [47].

Importantly, and as a final point for this section, it is important to highlight that in addition to its roles in muscle recovery and mitochondrial biogenesis, mTOR is also pivotal in modulating immune responses. Its signaling pathways can influence the growth and function of immune cells, thereby affecting the body’s inflammatory response and tissue repair mechanisms following physical exertion. This dual role of mTOR provides a crucial link between post-effort recovery and immune system adaptations. Understanding this connection is essential for comprehending the mechanisms of MI discussed later. For instance, the review from Jones and Pearce suggests that targeting mTOR signaling offers potential strategies for controlling immunity in various diseases, by altering the metabolic programming of tissue-resident immune cells [41]. As an example, both rapamycin treatment and genetic deletion of mTOR have been demonstrated to suppress the differentiation of helper T-cells (Th)1, Th2, and Th17 effector T-cells, while simultaneously enhancing the differentiation of regulatory T-cells (Treg) [48]. mTOR inhibitors are currently utilized in clinical settings as immunosuppressive and anticancer agents [49]. Additionally, they are being developed for various other purposes, including the rejuvenation of tissues like the immune system. Thus, a proper modulation of this pathway could be of paramount importance for the “metabolic flexibility” of the immune cells, which is especially affected post-effort, and could thus be viewed as an interesting feature in a recovery protocol after strenuous exercise [50].

### 4.2. Peroxisome Proliferator-Activated Receptor-Gamma Coactivator (PGC-1α)

Initially recognized as a coactivator of nuclear receptors in brown adipose tissue [51], PGC-1α not only regulates mitochondrial biogenesis but also significantly influences various mitochondrial functions beyond biogenesis [52]. It plays a critical role in mitochondrial quality control by modulating processes such as fission, fusion, and mitophagy. By overseeing these mechanisms, PGC-1α helps in maintaining mitochondrial health, which is crucial in addressing recuperation post-effort. Moreover, its regulatory effects on mitochondrial quality can impact insulin sensitivity, highlighting its potential importance in the mechanisms of hypertrophy, as well as metabolic health and metabolic disorders. In concert with mTOR, PGC-1α plays a pivotal role in mediating mitochondrial adaptations following resistance training. This transcription coactivator is stimulated by exercise and regulates the expression of mitochondrial metabolism genes, which are essential for efficient energy production and recovery [29,53,54]. Importantly, the elevation of PGC-1α post-exercise initiates mitochondrial biogenesis and contributes to the enhancement of muscular oxidative capabilities, thereby supporting recovery and adaptation after strenuous physical effort [54,55,56]. Finally, and in keeping with the eustress concept, it is interesting to mention that physiological (low, non-toxic) elevation of NO can promote mitochondrial biogenesis, largely by engaging pathways that converge on PGC-1α [57].

### 4.3. mTOR and PGC-1α Modulation for a Better Recovery?

mTOR and PGC-1α appear to be related as slight modulations of both the mTOR and PGC-1α pathways could enhance recovery following strenuous physical effort by improving mitochondrial function and reducing muscle damage. Inhibiting the mTOR pathway has been shown to promote autophagy and decrease inflammation, which are essential for muscle repair after intense exercise [34]. In addition, while well known for its antioxidant effects, research indicates that curcumin exhibits mTOR inhibitory effects, which have been associated with reduced muscle damage and improved endurance in animal models [58]. It is important to note that findings from animal models may not always directly translate to human physiology, and the effects observed with curcumin’s mTOR inhibitory potential require careful consideration when applying these results to human exercise recovery contexts. However, these studies suggest that moderating mTOR activity post-exercise could facilitate the recovery process by promoting cellular repair mechanisms rather than hypertrophy, which is primarily driven by unrestricted mTOR signaling. Moreover, the PGC-1α pathway is a critical regulator of mitochondrial biogenesis and energy metabolism. Mild modulation of PGC-1α activation can help balance energy expenditure and mitochondrial function post-exercise. Various studies highlight the importance of PGC-1α activity in improving muscle mitochondrial function and indicate that excessive upregulation can lead to oxidative stress, which may be counterproductive during recovery [59,60]. We, therefore, suggest that a controlled modulation of PGC-1α could facilitate mitochondrial adaptations while assisting in the overall recovery of muscle fibers following high-intensity efforts.

The interplay between mTOR activation and PGC-1α expression underscores the complex biochemical responses elicited by resistance training that collectively promote mitochondrial integrity and improve recovery outcomes [61]. Furthermore, the interaction between mTOR and PGC-1α suggests that strategic mild limitations on these pathways could optimize energy homeostasis, as studies indicate that inhibiting mTOR can enhance the activation of PGC-1α, thereby coordinating the adaptations necessary for recovery [60]. For instance, this interaction implies that post-effort strategies aimed at slight inhibition of these pathways can streamline the recovery process, enhance metabolic efficiency, and preserve muscle integrity by balancing the dual roles of growth and oxidative responses during recovery phases.

The interplay between mTOR inhibition and PGC-1α activation presents a notable avenue for therapeutic intervention: for instance, and even if not assessed in humans or within a sport-related context, rapamycin was reported to reduce RAPTOR, an mTOR complex component, while increasing PGC-1α and ATG13 levels in middle-aged mice [62]. This resulted in decreased mitochondrial oxidative stress and enhanced autophagy, leading to reduced age-related damage, such as lipofuscin accumulation. These changes may contribute to the drug’s ability to increase longevity by slowing down aging processes. This example of rapamycin’s ability to enhance autophagy and reduce oxidative stress suggests a promising avenue for exploring MI applications in post-exercise recovery, potentially leveraging these mechanisms to optimize cellular regeneration and resilience.

While current research provides substantial evidence for the roles of mTOR and PGC-1α pathways in enhancing recovery by improving mitochondrial function and reducing muscle damage, some aspects remain speculative. The precise mechanisms and optimal modulation strategies are not entirely understood, and much of the evidence is derived from in vitro or animal studies, which justifies cautious interpretation. This includes the extent to which mild modulation of these pathways can be harnessed for optimized recovery in humans, as individual responses may vary.

## 5. The Consequences of Oxidative Distress in Reducing Performance and Delaying Recovery

### 5.1. ROS, Performance and Recovery

Oxidative distress results from excessive ROS that overwhelm antioxidant defenses leading to cell damage, since various cellular components, including lipids, proteins, and nucleic acids, are affected by these high levels of ROS [63,64]. This cellular damage not only impairs muscle architecture but also disrupts intracellular signaling pathways, potentially leading to mitochondrial dysfunction and subsequently exacerbating ROS production, which in turn, may create a vicious cycle that ultimately hinders muscle performance and recovery [65,66]. Thus, oxidative distress can decrease performance and protract recovery.

The impact of oxidative distress extends beyond direct damage because high levels of ROS are closely linked to inflammation. Elevated ROS levels can trigger inflammatory cytokines release, which may amplify muscle protein degradation and inhibit regeneration processes [67,68]. For instance, conditions characterized by elevated oxidative distress have been shown to correlate with an increase in myostatin expression, which negatively regulates muscle growth [69]. Additionally, muscle fibers subjected to oxidative distress exhibit impaired insulin signaling, a critical pathway for nutrient uptake and metabolic health, which can further delay recovery from exercise-induced damage [3,70].

Exercise-induced muscle damage often manifests as delayed onset muscle soreness, driven by inflammatory responses that are initiated by oxidative distress. This process can result in prolonged muscle atrophy and could hinder the regenerative capacities of satellite cells [71]. In summary, the consequences of oxidative distress are multiple; they encompass immediate muscle damage, but also long-term functional impairments through chronic inflammation and metabolic dysregulation, and slowing down the recovery process.

Addressing the quantitative impact of oxidative distress on performance and recovery would be interesting in the context of this review but it presents numerous challenges due to the variability in influencing factors. The timeline and magnitude of these effects are highly dependent on several variables, including the type of exercise performed, the specific population being tested, and the physiological markers being studied. Additionally, the effects can differ significantly based on whether they are localized or systemic. These nuances make it difficult to provide a uniform timeline or measure of impact. Various studies, including the work of Vezzoli et al., have discussed these limitations [72], highlighting the complexity involved in accurately assessing the role of oxidative distress across different contexts.

### 5.2. Post-Effort Windows for ROS Production, Oxidative Distress Management and Recovery

Intense exercise activity induces ROS production and, if the recovery period is too short, it can lead to excessive oxidative stress (or distress) and cell/organ damage [73,74]. Regarding the role of ROS in recovery (through their functions as signaling molecules) as well as their deleterious effects if accumulated for a long duration, a better comprehension of the temporality of their production and their delayed effects post-training is necessary. For the post-effort windows ranging from 8 to 72 h, an animal study examined the impact of varying recovery periods on protein synthesis in mouse skeletal muscles [75]. Mice underwent exercise with either 72 h, 24 h, or 8 h recovery periods, revealing that protein synthesis was enhanced in the 72 h and 24 h groups but not in the 8 h group. Although mTOR signaling was activated in all groups, the 8 h group also experienced increased oxidative distress, suggesting that short recovery periods are insufficient to enhance muscle protein synthesis effectively. When assessed within a shorter time frame post-effort, these results were confirmed in humans, in untrained people and in athletes. Indeed, the findings from Steinberg et al.’s study with sedentary participants indicated that exercise induced oxidative distress within the first 30 min of the recovery period following cycling exercise [76]. In these participants, this training protocol increased plasma thiobarbituric acid reactive substances (TBARS) and reduced both erythrocyte glutathione (GSH), and plasma ascorbic acid (RAA). The participants also experienced an increased cytokine response (interleukin (IL)-6 and tumor necrosis factor (TNF)-α), which occurred almost simultaneously in the blood. Again, another example of such a fast metabolic response to exercise was confirmed in experienced athletes, and within the context of training sessions focused on an extreme conditioning program aimed at boosting work capacity, featuring a combination of cardiovascular and muscular exercises conducted 24 h apart [77]. This regimen was assessed for its impact on cytokines, muscle power, as well as blood lactate and glucose levels in trained male Crossfit^®^ athletes, and revealed a significant increase in blood lactate, glucose and IL-6 without impairment in muscle power. Another study showed that extreme-conditioning sessions such as Crossfit^®^ triggered an immediate and strong oxidative distress response in the blood that was similar to that caused by a conventional session of high-intensity treadmill running [78]. It is important to notice, however, that most of the human studies assessing mTOR activation post-effort are not solely considering blood marker assessment but primarily focus on nutritional timing strategies to improve protein turn-over and recovery post-effort. For instance, in the context of recovery from endurance activities, studies on resistance-trained adults suggest that protein consumption around 0.25 g/kg per meal may not adequately compensate for exercise-induced leucine oxidative loss, especially following activities like treadmill running [79,80]. Again, regarding the nature of the exercise, the intensity, the population and lifestyle parameters, it is difficult to provide a fixed answer regarding the exact timing of the recovery period for optimizing protein synthesis. This finding highlights the complexity of human recovery processes and suggests that temporal windows for optimal protein synthesis observed in animal models, such as the distinct outcomes at 72 h and 24 h compared to 8 h recovery periods, might not directly translate to humans. Nonetheless, drawing inspiration from these results provides a foundation for developing tailored strategies that align with these temporal windows to optimize recovery and enhance protein synthesis in human subjects.

Thus, as a general conclusion for this section, it could be highlighted that, with the aim to support optimal recovery and well-being, coaches could consider avoiding the scheduling of high-intensity sessions with less than 24 h of recovery between them. Incorporating potent antioxidant or immunomodulatory strategies, which will be developed further in the review, could help in mitigating oxidative stress and enhancing immune function. Such an approach may provide the necessary adaptation time for athletes, promoting both physiological recovery and maintaining overall health.

### 5.3. Excessive ROS Production, Oxidative Distress Management and Recovery: The Role of the Immune System

As mentioned before and as nicely summarized by Wang et al., exercise-induced excessive oxidative distress impacts skeletal muscle tissue in two ways [81]. Appropriate exercise can stimulate the production of physiological levels of ROS, supporting the normal functioning of skeletal muscles and aiding in exercise adaptation. Conversely, overexertion may lead to an overproduction of ROS and NRS, causing excessive oxidative distress, and high NO levels, which can react with superoxide anions, leading to peroxynitrite production, potentially leading to protein nitration, which can result in fatigue and damage to muscle cells [82]. Therefore, managing ROS production could be beneficial for recovery by preventing cellular damage and supporting optimal muscle performance.

The immune system is closely linked to redox status, overall health, and ultimately recovery. Importantly, the increase in post-exercise ROS production is not exclusive to muscle cells but also occurs within the immune cell compartment. In their study, Suzuki et al. assessed the capacity of neutrophils to generate ROS, in samples retrieved from healthy volunteers having completed 90 min bicycling sessions at the identical absolute intensity, with a power output held at 90 W for three consecutive days [83]. In this study, the opsonized zymosan-stimulated chemiluminescence response showed a significant increase during the 60 min exercise session and remained elevated at least until 3 h post-exercise, attesting to an increased ROS production by these immune cells during and after the session.

Furthermore, beyond their pivotal role in muscle cells, managing the excessive ROS and NRS production and oxidative distress in immune cells during the post-exercise recovery windows could significantly enhance recuperation. This comprehensive perspective that recognizes the role of the immune system in managing oxidative distress highlights the benefits of a holistic approach to help recovery, optimizing performance and reducing injury risk. Thus, addressing post-exercise recovery through a multi-faceted approach such as MI that includes immune system support is crucial, and managing the excessive ROS/RNS production and oxidative distress in immune cells during the post-exercise recovery windows could significantly enhance recuperation.

As developed in the next section, the redox homeostasis can be maintained through several factors, some partly due to personal sensitivity due to genetics (with no external possibility for modulation), and others that could be externally controlled (lifestyle, nutrition, training regimen, and supplementation), all contributing to proper recuperation post-effort.

## 6. Factors Modulating Redox Homeostasis and Recovery Capacity

### 6.1. Inter-Individual Variability in Response to Exercise and Training Status

Redox homeostasis and lactate clearance are modulated by a plethora of factors, including the intensity of active-recovery protocols [84] and nutrition, which, in general but especially around the training windows, is crucial to ensure proper recovery after an intense effort. Indeed, a study has shown that acute exercise and high-glucose ingestion produce dynamic and highly individualized changes in systemic redox biomarkers in healthy men [85]. Following exercise, both oxidative stress and antioxidant defense markers generally increase, while the effects of high-glucose ingestion vary depending on the timing and the specific biomarker measured. Some markers rise, others fall, and some remain unchanged, highlighting substantial inter-individual variability in redox responses. These findings emphasize the importance of interpreting redox biomarkers within the context of the individual, the specific biomarker, and the nature of the physiological stimulus, suggesting that personalized approaches may be necessary for assessing and optimizing redox balance and related health outcomes. In the same approach, MacKeegan et al. explored the complex role of ROS in the context of exercise and insulin resistance [86], emphasizing that while moderate ROS production during physical activity is essential for normal cellular signaling and metabolic adaptation, excessive ROS can contribute to excessive oxidative distress and the development of insulin resistance. Indiscriminate antioxidant supplementation may blunt beneficial exercise-induced adaptations, arguing for a more personalized approach to antioxidant therapy. By considering individual differences in oxidative stress responses and metabolic health, their article suggested that tailored antioxidant strategies could optimize both exercise benefits and insulin sensitivity, reinforcing the fact that new therapeutic avenues are still needed in this field.

### 6.2. The Influence of Diet, Lifestyle Factors, and Genetic Polymorphisms

**Figure 1:**
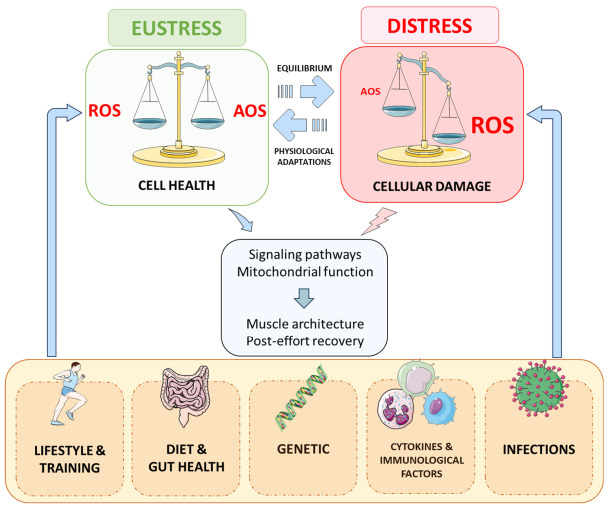
Recapitulative scheme of the dual role of ROS in the dynamic equilibrium between eustress and distress, and the main factors involved in post-effort recovery. The scheme illustrates the balance between ROS and the antioxidant systems (AOSs), which determines the redox status of the cell: eustress (green box), a state of physiological adaptation, necessary for cellular health, or distress (red box), a state of imbalance associated with cellular damages. Blue-dotted arrows represent the dynamic equilibrium (reversibility) between the two states of “physiological eustress” and distress, and that the state of distress can be reverted back to cellular eustress, which is needed for health and general evolution. Within the scope of this review, such equilibrium permanently impacts cellular pathways and mitochondrial function, ultimately leading to (grey arrow), at organ and organism levels, modulations in muscle architecture and post-effort recovery. The red flash symbol means that, when the distress state is maintained over a long period of time, and for too long, its consequences might impair cell health and promote maladaptation and diseases. Plenty of factors, illustrated as a non-exhaustive list of pictograms in the lower part of the scheme (yellow box: lifestyle and training, diet and gut health, genetics, cytokines and immunological factors, infections, etc.), can interfere and modulate the eustress/distress equilibrium, influencing the individual’s recovery capacity after an intense effort.

Having explored the complex interplay between various forms of physical training, individual training status, and recovery protocols on the antioxidant systems, it becomes evident that the body’s ability to counteract oxidative distress is influenced by numerous factors beyond just exercise. While physical activity can enhance the efficiency of the antioxidant systems through adaptive responses, it is essential to consider other external and internal elements that may impede these defense mechanisms. In particular, lifestyle choices such as diet and exercise, along with inherent genetic predispositions, play crucial roles in shaping the body’s oxidative balance. Understanding how diets, lifestyle factors, and genetic polymorphisms can either compromise or bolster the antioxidant systems is the next critical step in comprehensively evaluating an individual’s oxidative health. From a general health perspective, the imbalance between the production of ROS and the efficacy of antioxidant systems is a critical aspect of many chronic diseases, including neurodegenerative diseases, cardiovascular diseases, diabetes mellitus and chronic kidney diseases [13]. Genetic variants, particularly single nucleotide polymorphisms (SNPs) in genes encoding antioxidant enzymes such as superoxide dismutase (SOD), catalase, and glutathione peroxidase (GPX), can significantly impair the antioxidative potential and increase susceptibility to oxidative stress-related conditions [87,88,89]. For instance, SNPs in the catalase gene, such as the C-262T variant, have been suggested to lead to decreased enzyme activity, thereby compromising the detoxification of harmful H_2_O_2_ [90]. Furthermore, dietary habits significantly influence antioxidant levels; a diet rich in unhealthy fats can exacerbate oxidative distress and elevate ROS production, further straining the antioxidant systems [91]. The direct repercussion of such diets on mitochondria health has been studied by Vial et al., as their in vivo model of rats fed with a high-fat diet allowed them to observe a reduction in the mitochondrial quinone pool and a significant alteration in the composition of mitochondrial lipids [92]. These alterations seem to fundamentally contribute to the inhibition of fatty acid oxidation and mitochondrial OXPHOS, while also causing an increase in mitochondrial ROS production. Interestingly, as nicely summarized by Morita et al., caloric restriction downregulates mTOR signaling, leading to selective translation of mRNAs encoding mitochondrial regulators, which may contribute to lifespan extension [93]. This suggests that under nutrient deprivation, mTOR signaling can shift the balance toward mitochondrial maintenance and efficiency. Notably, numerous components of the electron transport chain complexes, such as NDUFS6, ATP5D, ATP5L, and ATP5O, which exhibit changes in expression during the aging process, have been demonstrated to be under translational control by mTOR. Additionally, it was shown that mTORC1 enhances mitochondrial activity and biogenesis by selectively promoting the translation of nucleus-encoded mitochondrial mRNAs through the inhibition of eukaryotic translation initiation factor 4E (eIF4E)-binding proteins (4E-BPs) [94]. These data thus suggest that, regarding insulin sensitivity, which can be triggered by poor diet or lifestyle factors, a feed-forward loop connecting mRNA translation to OXPHOS, could also be highlighted, through aberrant mTOR signaling to abnormal cellular energy metabolism conditions. In addition, lifestyle factors such as sedentary behavior, smoking, and excessive alcohol consumption have also been linked to diminished antioxidant activity and increased oxidative distress [95,96]. Notably, obesity independently raises levels of lipid peroxidation and reduces the activity of protective enzymes such as erythrocyte CuZn-SOD and GPX, indicating that lifestyle interventions aimed at improving diet and increasing physical activity could enhance antioxidant defenses [91]. Consequently, the interaction between genetic predispositions and lifestyle choices forms a complex framework impacting the antioxidant systems. This interplay makes certain individuals more vulnerable to diseases associated with oxidative distress, depending on their genetic makeup and lifestyle [88,97]. While genetic testing for SNPs in key antioxidant enzymes like SOD and CAT provides valuable personalized information, our review focuses on the need for targeted therapeutic interventions, such as MI, that address the resulting functional impairment of the redox system rather than only the genetic predisposition. This multifactorial approach holds critical implications for creating personalized strategies aimed at mitigating oxidative damages through nutrition and genetic considerations. To summarize all of the above-mentioned inter-connections, Figure 1 illustrates the dual role of ROS in the balance between eustress and distress during exercise and recovery. When ROS production is balanced by antioxidant systems (AOSs), a state of eustress is maintained, promoting cell health and beneficial physiological adaptations. However, excessive ROS generation “overwhelms” antioxidant defenses, leading to distress, cellular damage, and impaired recovery. The figure also highlights how ROS-mediated signaling pathways and mitochondrial function influence muscle architecture and post-exercise recovery. Several factors contribute to this balance, including lifestyle and training, diet and gut health, genetic background, immunological factors, and infections.

### 6.3. Systemic Inflammation and Circulating Cytokines

Systemic inflammation and the release of pro-inflammatory cytokines are crucial modulators of redox homeostasis, profoundly affecting the balance between oxidative stress and antioxidant defense mechanisms. Inflammatory cytokines such as TNF-α, IL-1β, and IL-6 are known to trigger an increase in ROS production through the activation of immune cells, including macrophages and neutrophils [98,99]. This enhanced ROS generation not only promotes inflammation but also contributes to oxidative damage, establishing a feedback loop that exacerbates the inflammatory response [100,101]. For instance, TNF-α can activate NF-κB and, when signaling through tumor necrosis factor receptor 1 (TNFR1), can also lead to the activation of all three major Map Kinase signaling cascades, ERK, p38, and JNK [102]. Interestingly, the activation of these pathways can also promote further ROS production, creating a feedback loop. Thus, the interplay between systemic inflammation, cytokine release, and redox homeostasis is a pivotal aspect for understanding disease mechanisms and developing therapeutic strategies.

## 7. Antioxidant Systems

In the context of post-exercise recovery, cells employ an array of antioxidant systems to protect themselves from the potentially harmful effects of ROS accumulation, when generated during physical activity. These antioxidant systems include both (1) enzymatic and, (2) non-enzymatic components. Enzymatic antioxidants, such as SOD, catalase, and GPX, play a critical role in catalyzing the conversion of ROS into less harmful molecules, thereby mitigating oxidative stress. Non-enzymatic antioxidants, like vitamin C, vitamin E, and glutathione, act as “scavengers”, that can directly neutralize ROS. Together, these systems ensure cellular integrity and facilitate efficient recovery and adaptation following exercise by maintaining an optimal balance of ROS, which is crucial for signaling processes implicated in muscle repair and growth.

### 7.1. Antioxidant Supplementation for Post-Effort Recovery

To mitigate exercise-induced oxidative stress and inflammation, several strategies exist, including pre-exercise cooling, dietary supplements like polyphenols, sulforaphane-rich foods, and probiotics, alongside specific diets such as low carbohydrate, high-fat ketogenic diets [103]. These approaches could reduce inflammatory markers and oxidative damages, although potential adverse effects should be considered.

As an example, alpha-lipoic acid (ALA) is a naturally produced compound recognized for its strong antioxidant and anti-inflammatory effects [104]. Because it can function in both water-soluble and fat-soluble environments, it can penetrate the cytoplasm and mitochondria, which are the primary sites for ROS production during exercise. Thus, it is garnering increasing attention for its potential therapeutic applications in conditions associated with oxidative stress such as in the context of recovery after high-intensity training. Isenmann et al. suggest that supplementing with ALA during periods of intensive training may lead to decreased muscle damage and inflammation, alongside enhanced recovery [105]. In their study, the ALA group experienced a noticeable reduction in muscle damage and inflammation after a chronic training phase, as the IL-6 concentration in the placebo group was significantly increased compared to the ALA group. Additionally, back squat performance significantly declined in the placebo group, whereas it remained stable in the ALA group. Another study highlighted the importance of combining dietary supplementation with ALA and physical exercise, both pre- and post-effort, to boost fitness indicators and reduce recovery time for weightlifters [106]. A double-blind experimental study tested the effect of short-term ALA supplementation on knee extension-induced oxidative stress [107]. After oral supplementation with ALA, there was a noticeable increase in blood total antioxidant capacity. In contrast, in the group that did not receive supplementation, there was an increase in DNA damage, as well as an increase in lipid peroxidation and H_2_O_2_ following exercise.

A recent systematic review and meta-analysis synthesizing data from eight studies (188 subjects) found that mitoquinone, a mitochondria-targeted antioxidant, effectively reduced exercise-induced oxidative damage [108]. However, acute or chronic supplementation did not improve endurance exercise performance in healthy individuals, highlighting that, in terms of supplementation, discrimination should be made between the potential effects of antioxidants on the recuperation side, and on the performance side itself, even if both are linked. Finally, caution should be taken regarding antioxidant supplementation as there is emerging evidence from preclinical and recent human studies indicating that non-enzymatic antioxidant supplementation may have a blunting effect on the beneficial adaptations associated with exercise endurance training [109]. For instance, daily supplementation with vitamins C and E was shown to reduce markers of mitochondrial biogenesis from endurance training, however, without affecting VO_2_ max or running performance [110]. This suggests caution when combining antioxidant supplements with endurance exercise due to potential interference with cellular adaptations. Another example is the study from Ristow et al., which highlighted that antioxidant supplements inhibit the activation of key molecular regulators related to insulin sensitivity and the body’s own antioxidant defense mechanisms that are typically stimulated by physical exercise [111]. These authors propose that temporarily elevated oxidative stress levels may serve as a beneficial process, particularly concerning the prevention of insulin resistance and type 2 diabetes mellitus. This “eustress/distress barrier” has also been illustrated in Figure 1.

These findings challenge the traditional view of antioxidant supplementation as universally beneficial, suggesting that it might interfere with the natural adaptive processes induced by exercise. Acknowledging these controversial results highlights the complexity of antioxidant use in sports nutrition and underscores the need for a more nuanced understanding of its role in exercise adaptation. This perspective encourages a critical analysis of current evidence, recognizing the conflicting findings and limitations within existing research, and providing interesting perspectives for the use of cytokines as antioxidant system modulators.

### 7.2. Modulation of Antioxidant Systems Through Cytokines

The effects of antioxidant supplementation on oxidative stress and inflammatory cytokines are ambiguous, with outcomes influenced by factors such as age, sex, health status, and exercise protocol [112]. More research is still needed to clarify the role of antioxidant supplements in this context, considering individual and exercise-related variables.

As evoked in the previous paragraph, enzymatic antioxidant systems are of paramount importance for ROS control, and these systems also play a role in muscle maintenance, which is crucial for the longevity of athletes and the general population [113,114]. Indeed, the importance of SOD for recovery has been documented in vivo, as the knockout of CuZn superoxide dismutase (*Sod1KO*) results in increased oxidative stress, leading to faster muscle atrophy and weakness [115]. Cytokines and growth factors, due to their pleiotropic actions, are one possible way to modulate SOD expression and/or activity, as reported within the context of extra-cellular SOD (ESOD), which plays a major role in the antioxidant defense within the extra-cellular spaces. Indeed, while TNF-α was found to exert an intermediate depressive effect on ESOD expression in human dermal fibroblasts, tumor growth factor-β (TGF-β) markedly suppressed its expression [116]. Moreover, the same study showed that TGF-β also repressed the activity of MnSOD in fibroblasts.

Glutathione peroxidases (GPXs) are a class of antioxidant enzymes that contain selenium, under the form of selenocysteine, which are capable of reducing H_2_O_2_, utilizing glutathione as a cofactor. These enzymes can be induced in skeletal muscles and are known to rise in those muscle fibers that are actively engaged during consistent exercise routines. In fact, several studies have demonstrated that endurance exercise training can lead to a 20–177% increase in GPX activity within skeletal muscles [9]. The “rescued” antioxidant effects of GPX towards SOD have been documented by Xu et al., as overexpressing GPX4 enhanced mitochondrial respiration and decreased hydroperoxide production in *Sod1KO* mice [115]. Such data are interesting, as they could suggest that if one of the cellular antioxidant systems is impaired, their “redundancy” could be a leverage of action for therapy, or for efficient post-effort recuperation strategies.

Regarding GPXs, an interesting inverse correlation has been established between the expression of GPX2 and pro-inflammatory cytokines such as IL-1β, IL-6, and TNF-α, in a large panel of human head and neck squamous cell lines, potentially suggesting that the inhibition of these cytokines could be a possible leverage to trigger the expression of such antioxidants [117]. However, it is crucial to recognize the limitations of this context; the observed inverse correlation is based on studies using human head and neck squamous cell cancer lines, which differ significantly from a sports and recovery setting. The dynamics in cancer cell lines may not directly mimic those in healthy individuals undertaking physical exertion. As MI formulations employ ULD of these above-mentioned cytokines, they show promise in this regard as they might reinforce GPX expression by down-regulating pro-inflammatory cytokine levels. In addition, another example of the effect of cytokines on the antioxidant systems could be cited through a study conducted in the RA context [118]. Here, anti-TNF-α treatment effectively reduces lipid peroxidation, indicating a positive impact on antioxidant systems. However, it did not appear to influence protein oxidation or carbonyl stress levels. Consequently, while MI presents an intriguing strategy, additional research is still necessary to explore its applicability and effectiveness in non-diseased contexts such as exercise recovery.

### 7.3. Modulation of Antioxidant Systems Through Cytokines: The Potential of Ultra-Low Doses (ULD) of Cytokines to Reduce Inflammation

The application of ULDs of key pro-inflammatory cytokines, specifically TNF-α, IL-1β, and IL-6, within MI formulations, represents a promising strategy for mitigating systemic inflammation. This approach harnesses the principle of modulating immune responses through highly diluted, still biologically active components. Evidence supporting this concept stems from both in vivo and in vitro investigations. In an in vivo study, performed in a mouse model of rheumatoid arthritis, the administration of a MI medicine (MIM) employing ULD of TNF-α and IL-1β, among other active substances, reduced the levels of circulating TNF-α and IL-1β compared to the vehicle control group [119]. Further corroboration was obtained through an in vitro study involving human primary enriched monocytes. These cells, sourced from six healthy donors, were subjected to an inflammatory challenge using lipopolysaccharide. The subsequent application of one capsule of the same MI formulation, containing ULD of TNF-α and IL-1β, led to a significant decrease in the secretion of the three cytokines: TNF-α, IL-1β, and IL-6, when compared to the vehicle control group [120]. All studies included a proper vehicle control group. This highlights that the direct anti-inflammatory action observed in the MIM-treated group is driven by the active substances. Beyond this specific formulation, other MIMs, which also incorporate ULD of the three aforementioned cytokines (TNF-α, IL-1β, and IL-6), have been tested in vitro. These formulations consistently exhibited anti-inflammatory properties across various established immune models [121,122]. This collective preclinical evidence reinforces the broader potential of ULD used in MIMs to regulate inflammatory pathways and reduce systemic inflammation.

## 8. Cytokines and Growth Factors Employed at Ultra-Low Doses in 2LMIREG

In the dynamic field of sports science and post-effort recovery, understanding the molecular pathways involved in muscle adaptation offers crucial insights for optimizing strategies for optimal recuperation. During and after exercise, skeletal muscle undergoes a series of adaptive responses facilitated by complex molecular signaling networks. Central to these processes are critical pathways such as the mTOR, AMPK, PGC-1α and NF-κB, which regulate muscle protein synthesis, energy metabolism, and stress responses. In this intricate landscape, cytokines and growth factors emerge as pivotal bioactive mediators able to influence all above-mentioned pathways.

**Figure 2:**
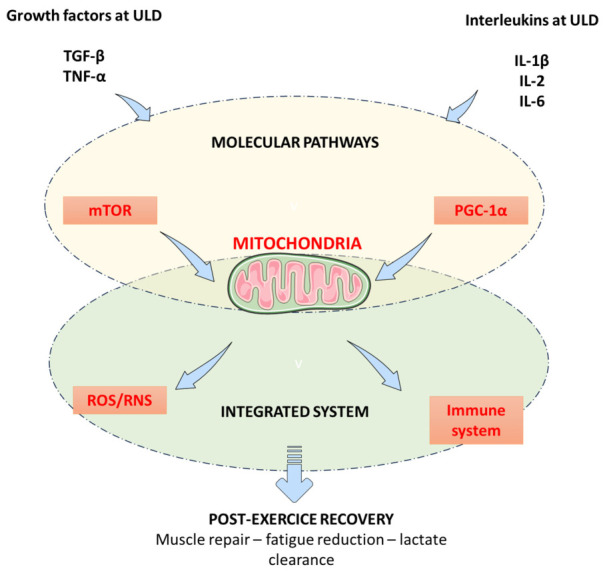
Recapitulative scheme of the relationships between some of the growth factors/cytokines, used at ultra-low doses (ULD) in the MIM 2LMIREG, the mTOR/ PGC-1α pathways, and the integrated systems involved in the proper management of post-exercise recovery (overall redox balance (ROS/RNS) and immune system). The mitochondria regulation appears as the core element of these systems. The blue arrows symbolize the connective links between the different elements of the system.

When employed strategically, cytokines and growth factors can act on the two sides of the muscle repair and adaptation processes, (1) the overall redox balance (ROS/RNS) and (2) the immune capabilities, through their potential to influence and modulate a plethora of molecular pathways, including mTOR and PGC-1α. Furthermore, by modulating inflammatory responses and cell communication, cytokines and growth factors may enhance recovery processes post-exercise, reduce muscle damage, and foster a resilient adaptation process, positioning them as promising agents in sports recovery strategies. In this section, the effect of several cytokines and growth factors on the above-mentioned pathways, mitochondria metabolism, and the connection between them will be discussed. Figure 2 summarizes the putative mechanisms through which ULD of growth factors and interleukins contained in the MIM 2LMIREG formulation may support post-exercise recovery. Growth factors such as TGF-β and TNF-α, together with ILs including IL-1β, IL-2, and IL-6, are proposed to modulate key molecular signaling pathways, notably mTOR and PGC-1α (yellow ellipse within the scheme). These pathways converge on mitochondrial regulation, which appears to represent the central hub of the system. Through its effects on mitochondrial function, the formulation may contribute to the coordination of redox balance (ROS/RNS regulation) and immune system activity within an integrated physiological response (green ellipse within the scheme). Altogether, these interconnected mechanisms could promote muscle repair, reduce exercise-induced fatigue, and enhance lactate clearance during the post-exercise recovery phase.

Specifically, a particular emphasis will be placed on (i) the two growth factors TGF-β and TNF-α, (ii) as well as the interleukins, IL-1β, IL-2, and IL-6, employed at ULD in 2LMIREG.

### 8.1. Growth Factors: TGF-β and TNF-α

Transforming growth factor-β plays a significant role in mitochondrial physiology and the mTOR pathway, intricately influencing cellular metabolism and energy homeostasis. TGF-β signaling has been shown to impact mitochondrial dynamics, including biogenesis, respiration, and apoptosis, thereby affecting the overall mitochondrial function and bioenergetics. In their study on human peripheral and tumor-associated lymphocytes, Dimeloe et al. reported that TGF-β significantly reduced the ATP-coupled respiration of CD4^+^ T-cells, and directly inhibited the activity of mitochondrial complex V (ATP synthase) [123]. Furthermore, solely inhibiting ATP synthase was enough to reduce IFN-γ production by CD4^+^ T-cells. These findings indicate that TGF-β directly affects T-cell metabolism, thereby impairing T-cell function through metabolic disruption. Furthermore, TGF-β interacts with the mTOR pathway, and the crosstalk between TGF-β and this pathway can modulate OXPHOS and ATP production, thus influencing cellular responses to metabolic stress. In their review on the role of TGF-β on muscle damage and regeneration, Kim et al. suggested that the biological responses to muscle damage, caused by eccentric muscle contractions (such as inflammation, regeneration, and fibrosis) are interconnected rather than independent [124]. In addition, a wide range of in vitro models other than muscle cells have been employed to document the interaction between TGF-β and the mTOR pathway. For instance, epithelial-like mammary gland cells exposed to TGF-β consistently exhibited an increase in size, ranging from 10% to 30%, and such treatment also activated the mTOR pathway [125]. Interestingly, in primary human lung fibroblasts, PI3K/mTOR signaling played a crucial role in TGF-β1-induced collagen synthesis [126], and TGF-β exposure was also shown to lead to the mTOR-dependent activation of gene expression programs that promote amino acids and glucose metabolism as well as OXPHOS [127]. In this study, mTOR was shown to be essential for the expression of ATF4 target genes, enhancing the expression of glycolytic enzymes, and the subunits of the mitochondrial electron transport chain.

Regarding inflammation, the study from Nam et al. investigated the effects of TGF-β1 on PGC-1α expression and mitochondrial dynamics in renal tubular epithelial cells [128]. TGF-β1 treatment led to decreased levels of PGC-1α transcripts and proteins and reduced mRNA expression of mitochondrial fusion gene *mitofusin* and *Tfam*, while increasing the expression of the fission-related gene dynamin-related protein 1 (*Drp1*). Additionally, TGF-β1 activated the nod-like receptor (NLR) protein (NLRP)-3 inflammasome, evidenced by higher levels of related genes and proteins, and increased concentrations of IL-1β.

Muscle injuries commonly lead to irreversible damage, with TNF-α playing a role in tissue degradation [129]. A study using Wistar rats demonstrated that inhibiting TNF-α with infliximab reduced degradation, enhanced muscle hypertrophy, and improved muscle force post-injury [130]. These findings suggest that TNF-α blockade could aid in muscle restoration, offering potential recovery benefits by accelerating healing and preserving muscle function. TNF-α is also a marker of inflammation post-endurance effort: the impact of a 2.5 h running test on plasma levels of TNF was assessed in eight healthy young male participants, and a significant increase in this factor was observed 1 h after the end of the running test [131]. In their studies, Ostrowski et al. reported that TNF-α plasma levels increased by 2- [132], and 2.3-fold compared to pre-race levels [133]. The average plasma concentration reached its peak immediately following the race, and then gradually decreased throughout the rest period, still remaining significantly elevated compared to the pre-race value up to 2.5 h after running. These findings, which validate that TNF-α inhibition, can aid muscle restoration, suggesting a clear therapeutic objective of reducing excessive TNF-α levels. It is important to note that while monoclonal antibodies like infliximab achieve this through pharmacological blockade, ULDs of both TGF-β and TNF-α are hypothesized to act through a fundamentally different mechanism: exerting a hormetic response to mitigate excessive cytokine levels and restore homeostasis. This approach seeks to regulate the systemic inflammatory response rather than performing a direct antagonism, offering a potential path to down-regulate these factors post-effort.

### 8.2. Interleukins: IL-1β, IL-2, and IL-6

Interleukins are mainly known for their immunoregulatory roles, but emerging research has highlighted their involvement in oxidative stress regulation. As developed further in this sub-section, certain ILs possess antioxidant properties and can modulate the body’s response to the oxidative distress generated during intense physical activity. By influencing inflammation, promoting the expression of antioxidant enzymes, and stabilizing cellular environments, ILs help balance ROS levels, thereby facilitating recovery and minimizing muscle damage. Understanding the intricate roles of these signaling molecules in mTOR pathways, and oxidative distress management, opens up new avenues for enhancing recovery strategies and ensuring sustained high performance.

First of all, and as shown by Ostrowski et al., IL-1β is a pro-inflammatory cytokine which greatly increases after strenuous exercise. When assessed immediately after a marathon, the plasma levels of IL-1β increased between 1.5- and 2.1-fold compared to the pre-race values [132,133]. From an immune perspective, an interesting link can be made between IL-1, mTOR and Th17 cells. Indeed, in their study, Gulen et al. observed that the proliferation prompted by IL-1 was eliminated in mTOR-deficient Th17 cells, highlighting the crucial role of mTOR activation in these cells [134]. Other in vitro models confirmed the importance of IL-1 for mTOR activation, as IL-1β was also proven to activate the phosphatidylinositol 3′-kinase (PI3K)/Akt/mTOR pathway in hippocampal neurons [135]. From a signaling standpoint, in CD4^+^ cells, the B-cell adapter for the PI3K (BCAP) adaptor has been shown to play an essential role in the activation of mTOR [136]. Indeed, BCAP transmits critical signals downstream of IL-1 and IL-18 receptors in CD4^+^ T-cells during the priming stage, thereby enhancing Th17 and Th1 cell responses, respectively. Interestingly, BCAP was shown to activate the PI3K/Akt/mTOR pathway downstream of IL-1β signaling in T-cells, leading to mTOR-driven increases in glycolysis. Of note, the involvement of mTOR in glycolytic processes parallels the fact that the mitochondria’s affinity for the primary mitochondrial substrate, ADP, varies significantly between low-twitch (oxidative) and fast-twitch (glycolytic) skeletal muscles [137], possibly highlighting here a difference in the role of IL-1β in the regulation of glycolysis in fast-twitch fibers especially. On the other hand, it seemed that IL-1β could also have inhibitory effects on mitochondria-related pathways, as the findings from Huang et al. demonstrated that both IL-1β and TNF-α led to a significant reduction in the expression of peroxisome proliferator-activated receptor gamma (PPARγ) and PGC-1 in vitro, as well as the DNA binding capacity of PPARγ [138].

In immune cells, IL-2 has been shown to influence the mTOR pathway either in innate immunity or in adaptive immunity. Indeed, this IL has been shown to activate the mTOR pathway both in T-cells and in natural killer (NK) cells [139]. In B-cells, it has been reported that IL-2 from Th cells enhanced extrafollicular plasma cell growth and low-mutated immunoglobulin (Ig)G secretion, and that it activates mTOR and influences metabolic pathways, acting as a key switch in B-cell fate between plasma and germinal center differentiation [140]. In addition, it has been shown that engaging the IL-2 receptor can prevent the onset of anergy in T-cells activated without co-stimulation, by inhibiting the expression of genes that induce anergy [141]. This effect was mediated by mTOR activation and possibly other signaling pathways, influencing a transcription factor pattern that blocks the nuclear factor of activated T-cells (NFAT)-dependent, activator protein-1 (AP-1)-independent gene expression responsible for reducing T-cell receptor (TCR) signaling and cytokine production in anergic T-cells. From a mechanistic standpoint, Fallone et al. hypothesized that IL-2 could activate mTORC1 through the JAK-signal transducers and activators of transcription (STAT) pathway [139]. In terms of recovery, it is essential to consider how IL-2 influences both oxidative distress and mTOR. Studies have shown that following physical exertion, pro-inflammatory cytokines such as IL-2 may exacerbate the production of ROS, which in turn can lead to cellular dysfunction and apoptosis [142]. Interestingly, soluble IL-2 receptor plasmatic concentrations were significantly elevated 1 and 2 days after exercise, when measured in healthy young males after the completion of a running test [131], possibly highlighting, again, the potential of lowering IL-2 as an enhanced-recovery strategy post-effort.

Regarding IL-6, its role in exercise and post-exercise is well-known, especially after long endurance training, when it has been shown to be significantly increased [103], even reaching 130-fold increases in the plasma after strenuous exercise, such as a marathon race, for instance [133]. To give a specific example, plasma IL-6 levels rose from 1.5 pg/mL prior to a marathon race to 94.4 pg/mL immediately after, then decreased to 22.1 pg/mL two hours post-exercise [132]. In their study, Nowak et al. evaluated the transcriptional changes in specific genes that encode receptors for chemokines and ILs in young, physically active men, aiming to gain insights into the immunomodulatory effects of physical activity [143]. Interestingly, a notable rise in IL-6R was observed one hour post-effort, irrespective of the participants’ exertion levels or age, suggesting a lactate recovery effect facilitated by the immune system. During contraction, skeletal muscle cells themselves are a source of IL-6 mRNA expression and protein release [144,145]. Therefore, IL-6 was referred to as a myokine and was considered to support energy supply and endurance performance [146]. Interestingly, in their in vivo study, Tominaga et al. added that muscle-infiltrating macrophages might also be a type of cell responsible for producing IL-6 during endurance exercise [147]. Regarding the link between this cytokine, mTOR and PGC-1α, IL-6 has been proven to reduce the expression of the mTOR-inhibitor regulated in development and DNA damage-response 1 (REDD1) in a STAT3-dependent manner, further leading to mTOR pathway activation [148]. In their model of septic mice, Yang et al. indicated that IL-6 deficiency mitigates skeletal muscle atrophy by reducing mitochondrial ROS production, achieved through the upregulation of PGC-1α expression [149]. Thus, strategies aiming at reducing IL-6 levels post-effort could help in recovery management, notably by supporting the upregulation of PGC-1α and reducing mitochondrial ROS production. Antioxidant strategies, which counteract oxidative distress, could therefore be beneficial by promoting more efficient recovery post-effort, helping to manage inflammation and optimize the recovery process before another bout of exercise. For instance, a single-blind, placebo-controlled study reported that four weeks of oral supplementation with a combination of vitamin C (500 mg/day) and vitamin E (400 IU/day) significantly reduced the release of IL-6 from active muscles, as well as the plasma IL-6 and cortisol levels, in response to three hours of dynamic, two-legged knee-extensor exercise at 50% of maximal power output, when compared to a placebo [150].

As discussed here, IL-1β, IL-2, and IL-6 play pivotal roles in the regulation of immune responses, and oxidative stress pathways, making them suitable targets for ULD-based therapies. The molecular mechanisms discussed translate to recovery enhancement through several modulatory effects, explained in the following sentences. While activating the PI3K/Akt/mTOR pathway to drive glycolysis in immune cells, IL-1β can also inhibit mitochondria-related pathways by reducing the expression of PGC-1α. Lowering IL-1β might be beneficial to recovery after intense exercise by balancing energy metabolism in muscle fibers and preserving mitochondrial health. Elevated IL-2 may exacerbate ROS production and cellular dysfunction. Strategic reduction of IL-2 levels is positioned as an enhanced-recovery strategy that could mitigate the cellular damage associated with the high ROS levels. While IL-6 acts as a myokine to support energy supply during exercise, sustained high levels post-effort contribute to inflammation and potentially skeletal muscle atrophy. Strategies aiming at reducing IL-6 post-effort could promote recovery by mitigating skeletal muscle atrophy through the upregulation of PGC-1α expression and reducing mitochondrial ROS production.

Thus, utilizing ULDs of IL-1β, IL-2, and IL-6 aims to exert controlled modulation of these signaling molecules. As this strategic fine-tuning intervention might enhance post-effort recovery, it is important to be aware that while current evidence supports the role of interleukins in modulating immune responses and oxidative stress pathways, the clinical outcomes directly related to recovery are not as well-documented. Future research should prioritize rigorous clinical trials focusing on measurable recovery outcomes, such as muscle soreness reduction, performance restoration, and biomarkers of muscle damage, to substantiate these theoretical claims. By correlating molecular interactions with tangible recovery benefits, such studies would provide a comprehensive understanding of how the use of ILs at ULDs can be effectively integrated into therapeutic strategies for enhancing post-exercise recovery. Until then, the potential of ULD ILs remains a promising yet unproven frontier in sports medicine and recovery science.

## 9. Micro-Immunotherapy: A Targeted Immunoregulatory Adjuvant Therapy Employing Cytokines and Growth Factors at LD and ULD That Could Help Recovery Processes

### 9.1. An Introduction to Micro-Immunotherapy Medicines (MIMs) and Their Specificities

In the realm of post-effort recovery, it is worth mentioning the development of the “hormesis” concept, which suggests that moderate exercise may play a role in enhancing antioxidant defenses [82]. Additionally, it has been proposed that cytokines, when utilized at ULD, may also contribute to the restoration of hormesis [151]. Indeed, the effects of MI can be explained by the hormetic dose–response curve, known for its non-linear pattern, observed when a stimulus is evaluated across a broad range of concentrations. The concept of hormesis may underlie cellular adaptation to these ultra-low responses, as it has been applied to diverse cellular functions such as proliferation, DNA repair, and, interestingly, for the scope of our review, in antioxidant responses [152]. Initially, hormesis described the cellular responses triggered by very low levels of potentially harmful agents as adaptive mechanisms that enhance the functionality and tolerance of biological systems to environmental stresses. These responses are evolutionarily conserved, broadly applicable across different organisms, and believed to result from complex integrative signal-transduction processes, resulting in a coordinated holistic response that can be transmitted to distant tissues or organs through a phenomenon known as “remote conditioning” [153]. Regarding the immediate systemic increase in pro-inflammatory cytokines after exercise (see previous paragraph), there is an opportunity to explore the use of MIMs as a novel approach in this area to further enhance recovery and immune responses after exercise. This section introduces MIMs, which are designed to modulate the immune system and cellular signaling processes by using signaling molecules, such as cytokines, hormones, growth factors, and nucleic acids at LD and ULD. In MIMs, the active substances ranging from 3 to 5 centesimal Hahnemannian (CH) dilutions are considered LD, and can orient the biological responses towards an activation, displaying immune-boosting effects, as documented for interferon (IFN)-γ (4 CH) in comparison with the vehicle used as a placebo [154]. Cytokines at ULD such as IL-1β (27 CH), TNF-β (27 CH), and IL-6 (27 CH) displayed anti-inflammatory effects and the capacity to inhibit the secretion of IL-1β, TNF-α, and IL-6 in vitro [119,120,121,122]. The hypothesized mechanisms of action of these ULD-based activities has been proposed in a study in which the presence of sub-micron particles in 27 CH unitary MI products were reported through qNano analysis [155]. The authors discussed the hypothesis that sub-micron structures are formed during the manufacturing processes of MIMs, which carry the starting material able to act as a biological signal and promote adaptative mechanisms in a non-linear dose–response, as hormesis.

Interestingly, Taherkhani et al. also discuss several cytokines in the context of physical activity and antioxidant supplementation [112]. Interleukin-1β, IL-6, and TNF-α are highlighted as key pro-inflammatory cytokines that increase in response to intense or unaccustomed exercise, contributing to muscle damage and inflammation, while regular moderate exercise can help modulate their levels and support immune regulation. IL-6 is noted for its dual role, acting as both a pro- and anti-inflammatory cytokine depending on the context, with its levels rising during exercise to help regulate the inflammatory response and promote recovery. While the immediate effects of intense exercise on pro-inflammatory cytokines are evident, understanding the underlying mechanisms and the potential of MIM in this context offers valuable insights. This is where emerging research on such compounds becomes particularly relevant, as they might hold the key to mitigating exercise-induced inflammation and oxidative stress. One such promising candidate is the investigational medicine MIM 2LMIREG, which has demonstrated interesting properties in our preclinical studies.

### 9.2. Preclinical Evidence on 2LMIREG Shows Antioxidant and Immunomodulatory Effects: An In Vitro Proof-of-Concept

In the context of post-effort recovery, it is interesting to specifically mention our preclinical data on the MIM 2LMIREG, which displayed antioxidant properties by lowering the generation of ROS in immune cells (specifically B-cells and neutrophils), and immunomodulatory effects, when compared to the vehicle capsules, used as a placebo control [156]. These findings, while preliminary, suggest that this medicine could contribute to the regulation of oxidative distress, and to physiological adaptation to maintaining immune homeostasis and preventing cellular damage. As it would be interesting to discuss the effect of MI on nitrosative stress, it is worth mentioning that the implications of ROS regulation by 2LMIREG on other molecular markers, such as iNOS/NOS2, would remain another area of investigation. iNOS is crucial in the production of NO, a compound that can contribute to nitrosative stress when overexpressed, especially following intense exercise. It would be intriguing to further explore whether 2LMIREG’s ability to modulate ROS overlaps with a regulatory effect on iNOS expression, thereby potentially mitigating the risk of nitrosative distress post-exercise. This understanding could have significant implications for improving recovery and reducing inflammation-related damage in physically active populations. The antioxidant properties were also observed in two colorectal cancer cell lines, HT-29 and SW620 [157]. This MIM could reduce ROS production by approximately 40% in both cell lines after 20 days of treatment, compared to the vehicle-treated cells. Furthermore, beyond its antioxidant capabilities, this MIM also exhibited anti-inflammatory properties, which can be beneficial for improving recovery processes. This was evidenced by the medicine’s ability to slightly reduce the expression of human leukocyte antigen (HLA)-DP in human peripheral blood mononuclear cell (PBMC)-derived M1 macrophages, when compared to the vehicle capsule used as a control. HLA-DP is a major histocompatibility complex class II molecule, whose expression is often upregulated on M1 (pro-inflammatory) macrophages. A reduction in HLA-DP expression suggests a down-modulation of the pro-inflammatory phenotype associated with M1 activity. This finding is particularly relevant in the context of physiological adaptation and recovery, especially in tissues undergoing repair, such as muscle. Indeed, to put our results into perspective, it is interesting to mention Voskoboynik et al.’s work, which presents consistent patterns across 75 examined studies, emphasizing exercise’s role in orchestrating a balanced and temporally regulated interplay between pro-inflammatory M1 and “reparative” M2 macrophage activity [158]. They suggest that exercise-induced health benefits are closely tied to this dynamic balance. Although their cited studies do not specifically address exercise-induced muscle damage or athletic populations, they propose a broader mechanism where an imbalance between pro-inflammatory and repair responses could potentially lead to disease development. In the context of exercise-induced inflammation, which physiologically differs from pathological inflammation, it is reasonable to hypothesize that similar effects might occur, facilitating recovery through the M1-to-M2 transition. Further studies are necessary to directly examine these dynamics in exercise-specific scenarios, especially regarding the reduction of HLA-DP in M1 macrophages and its implications for muscle recovery. However, the critical shift in macrophage phenotype during the successful resolution of inflammation and the promotion of tissue repair is already well documented. Macrophages transition from the initial pro-inflammatory M1-type, which is essential for pathogen clearance and debris removal, toward the M2-type resolving macrophages [159,160]. Indeed, M2 macrophages are characterized by the production of anti-inflammatory and pro-resolving cytokines, notably, TGF-β and IL-10, both crucial in accelerating the resolution of inflammation, promoting tissue remodeling, and fostering processes such as muscle growth and repair.

In addition, this MIM was also shown to exert a protective effect in a model of human primary macrophages, reducing by about 30% the dying/apoptotic cells from methoxy propyl acetate (PMA)/ionomycin-induced cell death, in comparison with the vehicle used as a control. In addition, when used to treat human PBMCs for 48 h, and under PMA-ionomycin stimulation, it was able to modulate the secretion of cytokines in a manner that would correspond to a Th17 signature [156]. This finding is relevant because the mTOR complex is involved in the expansion of committed Th17 cells [161], and emerging evidence connects Th17 cell metabolism to redox status, especially in the context of physical exertion and recovery in sports. Th17 cells are known for their roles in immune response and inflammation, and their metabolic profiles may be influenced by redox states. The differentiation and metabolism of Th17 cells are linked to redox homeostasis. Indeed, research by Zhao et al. highlights the p38 regulated/activated protein kinase (PRAK)-NF-E2-related factor 2 (PRAK-NRF2) axis as crucial in Th17 cell differentiation, showcasing its role in maintaining redox balance and aiding in glycolytic adaptations within these cells [162]. This is particularly relevant in post-exercise recovery, where oxidative stress often increases due to strenuous physical activity. Furthermore, Fu et al. demonstrate that increased oxidative metabolism and the production of ROS can inhibit Th17 cell differentiation, indicating that redox status directly influences the abundance and function of these cells during recovery from exercise-induced oxidative stress [163]. Additionally, aerobic glycolysis, a metabolic process that activates Th17 cells, generates ROS, which facilitates the relationship between metabolism and oxidative stress. This association was corroborated by linking high glucose intake to exacerbation of autoimmune conditions through ROS-mediated activation of TGF-β, thus directly connecting metabolic dysregulation and Th17 cell activity to oxidative stress pathways [164]. Understanding this metabolic reconfiguration is essential as it informs approaches for managing immunity and recovery post-exercise by regulating ROS levels. Finally, the study by Yang et al., highlighted that the glycolysis played a critical role in supporting the effector functions of lung-resident CD4^+^ Th17 tissue-resident memory cells [165]. This highlights glycolysis as a crucial metabolic pathway in the immune response within the lungs, which could be of particular interest during and after physical exertion, where enhanced metabolic demands and immune challenges are common. The Th17 pathway’s involvement in glycolytic adaptation is increasingly recognized as a novel concept within sports medicine. During recovery, the metabolic shift involving glycolysis likely provides Th17 cells with the necessary energy and biosynthetic precursors to maintain their effector functions. This supports the cells’ ability to contribute effectively to immune surveillance and tissue repair, underscoring the interplay between metabolic processes and immune function, paving the way for interventions and therapies, such as MI, aimed at improving recovery through targeted metabolic and immunological pathways.

It is important to mention that the results on Th17 cells and the effect of 2LMIREG were obtained under PMA/ionomycin stimulation, which does not fully reflect cellular and physiological reality, especially in the context of post-effort recovery. Nonetheless, these findings represent a small advancement and can guide further research on the impact of 2LMIREG on these cell populations, as further discussed in the next section. In practical terms, antioxidant strategies in sports may modulate Th17 cell activity and oxidative stress balance. The regulation of these cells through metabolic, dietary, and cytokine-based interventions could be a vital strategy in sports for optimizing recovery and immune function.

### 9.3. Translational Perspectives and Future Research Needs

The theoretical framework presented herein focuses on the potential of MIMs like 2LMIREG to help post-exercise recovery. Future human clinical studies are necessary to establish the ideal timing of intervention, (e.g., pre-effort, immediate post-effort, or sustained recovery phase), and the duration of treatment required to achieve sustained effects on redox balance and immune functions, in the healthy general population. Finally, while not less important, monitoring strategies that can reliably monitor the efficacy of the MIM are ultimately needed. Research on valid non-invasive biomarkers is necessary to ensure that the desired shift from oxidative distress toward eustress occurs.

## 10. Conclusions

Reactive oxygen species (ROS), reactive nitrogen species (RNS), and oxidative and nitrosative stress are considered crucial elements that play dual roles within physiological processes, acting as both signaling molecules and toxic components. These chemically reactive molecules encompassing O2·−, ·OH, NO, and H_2_O_2_, are vital for functions such as host defense and muscle adaptation, but an imbalance in their concentration can lead to the onset and progression of chronic diseases. Thus, it is essential to regulate their production and understand the pathways responsible for their homeostasis. By refining the concept of oxidative stress, distinguishing between oxidative “eustress,” which promotes beneficial physiological adaptation, and oxidative “distress,” which is linked to cellular damage and disease, is of paramount interest in the context of post-effort recovery. The interplay between oxidative and nitrosative stress and redox status regulation in the post-physical exercise windows (in both strength training and aerobic effort), is highly complex, and this review aims at analyzing the beneficial adaptative effects of oxidative “eustress” and the detrimental ones induced by “distress” through the lens of mitochondria-related and immune system adaptations, within the context of post-effort recovery. Indeed, while regular, moderate exercise is known to promote redox homeostasis, caution is advised with intense physical activity owing to potential oxidative distress that may require targeted recovery strategies.

This review also highlights the central role of the mTOR and PGC-1 pathway in mediating muscular adaptations and mitochondrial health. These mechanisms are sensitive to multiple factors, such as exercise intensity, diet, lifestyle, genetic factors, and immune responses, including cytokines. Faced with this complexity, personalized approaches are warranted.

Finally, positioning immunomodulation as another strategic, rather than palliative tool underscores its potential to actively enhance recovery post-exercise. Rather than just alleviating symptoms, strategic immunomodulation could optimize the body’s immune response to accelerate healing and reduce inflammation. This approach supports effective post-recovery strategies by taking into account individual variations such as genetic and environmental factors, allowing for personalized interventions. By tailoring recovery strategies to individual needs, immunomodulation not only aids in faster recovery but could also help overall “health resilience”. In this light, MI may represent a promising avenue worthy of further clinical investigation. A focus was thus placed on the potential of such a strategy in this context. Specifically, the preclinical data on 2LMIREG show its potential as a targeted therapeutic intervention, as it lowered the generation of ROS in B-cells and neutrophils and acted on Th17 cells, thus potentially supporting metabolic adaptations in vitro. These interactions between antioxidant properties and immune regulation provide valuable insights into how 2LMIREG can contribute to maintaining immune balance and enhancing recovery processes, emphasizing its potential utility in therapeutic applications. An emphasis is specifically placed on several growth factors and ILs, playing a role in these processes, and used at ULD in MIMs. Through targeted therapeutic interventions, MI could offer a promising strategy to enhance recovery following intense physical effort, minimize oxidative distress, and support overall cellular health. Future research should focus on the development of muscle cell models to further investigate the effects of 2LMIREG, and the execution of clinical studies is required to evaluate these potential effects in a real-world context and bridge the gap between preclinical results and practical applications for post-effort recovery.

## Data Availability

No new data were created or analyzed in this study.

## Abbreviations

15-PGDH: 15-hydroxyprostaglandin dehydrogenase; ADP: adenosine diphosphate; ALA: alpha-lipoic acid; AOS: antioxidant systems; ATP: adenosine triphosphate; AMPK: AMP-activated protein kinase; AP-1: activator protein-1; ATF4: activating transcription factor 4; BCAP: B-cell adapter for PI3K; CH: centesimal Hahnemannian dilution; CS: citrate synthase; Drp1: dynamin-related protein 1; eIF4E: eukaryotic initiation factor 4E; 4E-BP1: eIF4E binding protein 1; ESOD: extra-cellular SOD; GPX: glutathione peroxidase; GSH: glutathione; HIF: hypoxia inducible factor; HLA: human leukocyte antigen; IFN: interferon; Ig: immunoglobulin; IL: interleukin; JNK: Jun N-terminal kinase; LD: low doses; MAM: mitochondrial-associated membrane; MI: micro-immunotherapy; MIM: MI medicine; MnSOD: manganese superoxide dismutase; mtDNA: mitochondrial DNA; mTHF: mitochondrial tetrahydrofolate; mTOR: mammalian/mechanistic target of rapamycin; mTORC1: mTOR complex 1; Myc: myelocytoma; NADH: nicotinamide adenine dinucleotide + hydrogen; NADPH: nicotinamide adenine dinucleotide phosphate + hydrogen; NFAT: nuclear factor of activated T-cells; NF-κB: nuclear factor kappa-B; NO: nitric oxide; NK: natural killer; NLR: nod-like receptor; NLRP: NLR protein; NOS: nitric oxide synthases; NOX2: NADPH oxidase 2; OXPHOS: oxidative phosphorylation; PBMCs: peripheral blood mononuclear cells; PGC: peroxisome proliferator-activated receptor-gamma coactivator; PGE2: prostaglandin E2; PI3K: phosphatidylinositol 3′-kinase; PMA: methoxy propyl acetate; PPAR-γ: peroxisome proliferator-activated receptor gamma; PRAK: p38 regulated/activated protein kinase; PRAK-NRF2: PRAK-NF-E2-related factor 2; PRPS2: phosphoribosyl-pyrophosphate synthetase 2; RAA: reduced plasma ascorbic acid; REDD1: regulated in development and DNA damage-response 1; RNS: reactive nitrogen species; ROS: reactive oxygen species; S6K1: S6 kinase 1; SCI: spinal cord injury; SIGIRR: single Ig IL-1-related receptor; SNP: single nucleotide polymorphism; SOD: superoxide dismutase; STAT: signal transducers and activators of transcription; TBARS: thiobarbituric acid reactive substances; TCR: T-cell receptor; TFAM: transcription factor A, mitochondrial; TGF-β: tumor growth factor-β; Th: helper T-cells; TNF: tumor necrosis factor; TNFR1: tumor necrosis factor receptor 1; Treg: regulatory T-cell; TSC1: tuberous sclerosis 1; ULD: ultra-low doses.
